# Medical Miscellanies

**Published:** 1817-05

**Authors:** 


### MEDICAL MISCELLANIES.

### THE PORTE FEUILLE, No. V.

OR,

DEPOT FOR DETACHED FACTS AND INTERESTING PHÆNOMENA, OCCURRING IN THE PRACTICE OF THE FRIENDS OF THE MEDICO-CHIRURGICAL JOURNAL.

"Ore trahit quodcunque potest atque addit acervo."

#### *Tinctura Lyttæ applied to a Syphiloid Ulcer.*

I BELIEVE every medical man is well acquainted with the efficacy of Tinctura Lyttæ as a rubefacient, but I am not so certain that every surgeon is aware of its good effect, when applied to ulcers; and, under this impression, I am induced to state, that I lately applied it to a painful gangrenous ulcer of the penis, which resisted a great variety of the usual remedies, such as common poultices, fermenting cataplasms, pulv. carbon. vinum opii, and tinct. myrrhæ, &c. The first application of the tincture produced a considerable degree of inflammation over the sound and diseased parts: in twenty-four hours the appearance of the sore was more favourable; and in forty-eight hours, it had a clean and tolerably healthy appearance. Before the application of the tinctura lyttæ, half of the glans penis was destroyed, and the sore was making a rapid progress between the cellular substance and body of the penis: it certainly arrested the progress, and changed the nature of the diseased action of the part. It is also worthy of remark, that before the application of it, the patient, for several days, complained much of violent shooting pains from the ulcer to the testicles and groin, but, in a few hours after the application of the tincture, the pains left him and never returned. The result of this case made more impression on my mind, in consequence of Mr. Abernethy's excellent directions, with respect to the constitutional treatment of local diseases, having been rigidly attended to; and in promoting which object, the pil. hydrarg. had been exhibited till it slightly affected the mouth, but did not appear to produce any good effect on the sore. The patient was a married man, and I have every reason to believe the sore was not venereal; but that it was occasioned by some morbid secretion communicated by the wife. The sore had such a formidable appearance a few days before the change described took place, that a surgeon who saw it declared it was cancerous. C.

---

### *Injury of the Head.* By R. M, Royal Navy.

" *December 28th.*—William Grant, ætatis 14, was knocked down by an empty cask, which, falling from the height of three or four feet, lacerated the scalp to about three inches in extent, transversely in the direction of the coronal suture. The pericranium was not detached, and no fracture could be discovered. The boy was stunned, for a few seconds, by the blow; but, on recovering, expressed himself as quite well, save a slight pain in the wounded parts. Head shaved, and wound dressed by adhesive plaster and bandages.

*December 29th, ten o'clock, a.m.* Rested well, and continues free from any pyrexial symptoms; pain of the wound as last evening: to have a laxative powder of jalap and cream of tartar. *Five o'clock, p.m.* Has had several loose stools since taking the medicines; complains, at present, of pain in the forehead, and cold shiverings. Pulse 115, and sharp to the feel; feet and ankles cold; starting of the tendons; talks incoherently; slight œdema of the left temple. Examined the wound, which adhered by the first intention. Bled him immediately from the temporal artery to twelve ounces, and applied sinapisms to the ankles. *Six o'clock, p. m.* Soon after the blood-letting, the temperature of the integuments of the cranium rose considerably; and the pulse sank to 95, with less sharpness. Full vomiting next took place. At present, he has a wild, inconsistent look; but answers questions rationally. Sinapisms removed and blisters applied to ankles. *Ten o'clock, p m.* Pulse 100, and small; tongue moist; feet cold. *Eleven o'clock, p. m.* Pulse 124, and sharp; articulation imperfect. Temporal artery opened again, and bled to twelve ounces. Soon afterwards, the pulse sank to 106; heat of the lower extremities returning: to have a weak solution of tartarized antimony in water, in divided doses. *Twelve o'clock, p. m.* The medicine, after taking the second dose, made him sick, and he vomited; pulse 92; perception and articulation more distinct.

*December 30th, three o'clock, a. m.* Found him asleep; respiration easy; pulse 95; skin dry, and of nearly natural heat: and when he awoke, he complained only of pain of his ankles, and of his head. This last, he says, feels as heavy as if it were *lead;* no stool since last evening: continue the mixture of tartarized antimony. *Eight o'clock, a. m.* The wound is disunited, has a livid appearance, and discharges thin matter. Retains the mixture; skin moist. Continue the medicines. *Six o'clock, p. m.* Has had one copious loose stool; inclines much to sleep; blistered parts dressed. Mixture continued.

From this time he gradually mended, without any other accident than the bursting out of the temporal artery, by which he suddenly lost twenty-four ounces of blood."

This case, on the second day, presents some curious and anomalous symptoms. The cold shiverings, coldness of feet and ankles, and subsultus tendinum, might and would have deterred many from the use of the lancet; but the salutary effects of this powerful measure are here very conspicuous, as it evidently checked at once, a rising train of morbid actions that would soon have produced fatal disorganization in the tender structure of the brain.—Edit.

---

### *Curious Train of Anomalous Symptoms.*

A young gentleman, of ruddy complexion and very plethoric, had contracted some slight gonorrhœal affection, for the cure of which he applied to an irregular practitioner, who gave him a solution of oxymuriate of mercury to take internally. This medicine he had continued about three weeks, when the reporter was sent for to see him. He was now perfectly as red as a boiled lobster, from head to foot. He had considerable cough, hoarseness, coryza, and fever. The pulse was very hard and quick, the head painful, and the chest oppressed. He was now bled and purged freely; after which, antimonials and neutral salts were exhibited. In three days the scarlet eruption began to disappear, and a new train of symptoms to develope themselves. Real jaundice came on, with deep-seated pain in the region of the liver; high fever; cough and delirium. Venæsection was again repeatedly employed, the blood throwing up a yellow buff. Blisters were applied to the seat of pain; strong calomel cathartics administered; and afterwards, five grains of blue pill thrice a-day. In four days, the hepatic inflammation was abated; and now the yellow colour of the body changed gradually to a blue, and he assumed a frightful appearance. This did not last more than two or three days, when the cuticle of the whole body began to desquamate; and in four days there was not a particle left on any point of the surface. His face, however, turned into one universal black crust, which blocked up the nostrils and eyes, so that he could scarcely breathe or see. There was now great irritative fever, with wandering delirium at night. He was also reduced almost to a skeleton. Decoct. cinchonæ with mineral acids. After the cuticle had been cast quite off, his tongue swelled so that he could scarcely articulate, or swallow drink for two or three days; and the head-ache was now so intense, that blisters were applied to the nape of the neck, and purgatives daily exhibited. The lingual swelling then declined, and a good appetite took place.

Several severe paroxysms of head-ache succeeded, but they were relieved by brisk purgatives. Indeed, the state of the bowels, the eye and the tongue, evinced, for a considerable time, a slight degree of hepatic torpor, if not obstruction. He has since gone a journey of one hundred miles, and is fast recovering; but still troubled with a cough. J.

---

### MISCELLANEOUS.

Quicquid agunt Homines.

### *To the Editors of the Medico-Chirurgical Journal & Review.*

GENTLEMEN,

HAVING had occasion to communicate with Dr. Jenner, on the subject of Vaccination, he wrote me an interesting letter in reply; and in stating his opinion of small-pox, as compared with the vaccine, he thus expresses himself. "Indeed, I consider the vaccine and variolous as having the same origin, the latter emanating from the former in the shape of a variety." On reading this part of the Doctor's letter, my mind was immediately impressed with the importance of ascertaining the foundation of such an opinion, as far as depended upon actual experiment on the cow. I was further induced to put it to the test, on finding that no recorded account existed, as far as I could ascertain, of such experiment having been performed. With this view, therefore, on the 3d of March, 1816, I took variolous matter from a child with the natural confluent small-pox, and on the morning of the 4th inoculated three teats of a cow put up to feed. The child I took the matter from died the following morning. On the 5th, the punctures were slightly inflamed. The 7th, punctures more inflamed than yesterday, and one of them had an elevated appearance, with slight induration. The 8th, two of the punctures had the appearance of pimples, with a little surrounding inflammation; the other not so much inflamed as yesterday. The cow manifested much uneasiness on pressure, and kicked violently on touching any part of the two inflamed teats. The 9th, the pimples were diminished in size, and the surrounding inflammation was almost gone; but the cow did not manifest so much pain as yesterday on touching the parts. The 11th, the pimples and inflammation much subsided, without any appearance of lymph or matter, and the parts bore pressure without giving any uneasiness. The 14th, the parts had simply the appearance of a puncture with a clean lancet, the inflammation entirely gone; but several small white and horny pimples were observed on different parts of the teats, which did not afford the least fluid on puncturing. On examining this cow minutely, together with a man belonging to the farm, who had been much accustomed to cows, we discovered the marks of previous ulceration, and the man assured me he had no doubt of this cow having had the cow-pox, which sufficiently accounted for the failure of my experiment. I again, however, repeated the inoculation, but this failed to excite the inflammation, leaving no other appearance than would have happened from a puncture with a clean lancet.

I then got a cow that, to all appearance, never had the cow-pox, being quite large with her first calf, and never having been milked, which an intelligent farmer allowed me to inoculate with small-pox matter. In this second experiment, I was careful to insert as much of the variolous matter as possible. The daily progress presented little worthy of remark: no inflammation was produced; the punctured parts were slightly elevated, and somewhat indurated on the fourth day; but the cow did not manifest any feelings of pain or uneasiness, on pressing the parts freely. At the end of seven days, the elevation and indurated feel of the parts were entirely gone, and the punctures just visible, without any particular appearance, as with the former cow.—I again inoculated with matter taken from a child a few hours before insertion, which also failed in producing any effect. It now occurred to me, that perhaps the want of success might be attributed to the state of the parts, rather than to the want of power in the matter, to excite diseased action. I was led to form such an opinion from the shrivelled, and partly insensible state of the teats, in both the cows I had tried the experiment upon, compared with the parts in full action, as in a cow giving milk. I therefore selected, for my third experiment, a young cow, after her first calf was taken from her, and, as far as could be ascertained, never having had the cow-pox.

March the 6th. I inoculated this cow with variolous matter taken from a child with the natural, distinct disease, by means of two punctures in the same teat, and care was taken to milk her daily from the other three, leaving the inoculated one untouched. The cow was seen every second day; and on the fourth day the teat was much enlarged, with an anasarcous feel and appearance, which I attributed to the want of milking, but without inflammation, or action of the punctured part. On the 8th day, the teat, although diminished in size, had still the same feel and appearance, without inflammation. On the 14th day, there was scarcely any mark of the teat having been punctured. On the 16th I repeated the inoculation, and filled a very large puncture with small-pox matter, taken from a child the day before: this last inoculation produced no visible effect, and at the expiration of fourteen days the puncture was scarcely visible.—I do not, certainly, feel myself justified in asserting, that these trials are conclusive on the subject; and if you think them worthy of a place in your Journal, perhaps some of your numerous readers may be induced to repeat the experiments, and communicate the result of some already made.—The substance of this paper was communicated to the National Vaccine Establishment in June last, and I received the thanks of the Board for the same.

I am, Gentlemen,

Yours, &c.

RICHARD WARREN COLEY.

Cheltenham, Feb. 25, 1817.

---

### DEGRADED STATE OF MEDICINE IN SPAIN.

Don Lopez Matéos, a Spanish physician, in a work on the "Philosohy of Legislation," has drawn a deplorable picture of the state of the medical profession in that ill fated country. We have not the book, but shall present our readers with some sketches from it, as drawn in a French Critique.

If, says the French Reviewer, the progress of literature and the sciences has been slower in Spain than in any other country of Europe, we are not to attribute this to want of genius or intellectual vigour in the inhabitants, but to a radical vice in the *civil institutions*, which by a strange fatality have, almost always, been in opposition to the real character of this magnanimous people. Laws, dictated by the most horrible despotism, and religious practices, enjoined under pain of death by the most monstrous fanaticism, have checked the developement of genius, without however, being able entirely to stifle the germs of those grand ideas, which justice and liberty engender and protect. What might not be expected from the *unrestrained* genius of those proud Castilians, who, in the midst of chains, have proved by a few literary *chefs d'œuvres*, and a multitude of heroic actions, that they could dispute the palm of glory, *in every thing*, with surrounding nations?

Why then does Medicine continue among the Spaniards in a state of infancy, bordering on barbarism? It is because the most noble and useful of professions is there regarded as a vile trade or contemptible occupation! It is because medical students, reduced by turns to mendicity or servitude, are classed with the apprentices of masons and shoe-makers! That man must be imbued with a more than ordinary philanthropy, who could dedicate genius or talent to a profession so unjustly, so unwisely dishonoured. That physician must be warmed by a generous devotion indeed to the cause of humanity, who could fly to the succour of such ingrates, or enlighten their minds by the results of his studies or experience! Such, however, is Don Matéos, whose work is an honour to himself, while it exposes the disgrace of his nation.

The recriprocal influence of the moral and physical characters on each other, is illustrated by some striking examples. Thus the immortal Archimedes, plunged in meditations the most profound, and tranquil amidst the tumult of arms and the horrors of massacre, feels not the impious blow of the barbarian soldier. Others, on the contrary, become paralysed; deprived of reason, or even of life, during the pronunciation of their sentence. We see the hypochondriac, the maniac, exist whole days, or even weeks, without nourishment; and bear without complaint, the most rigorous cold, the most overpowering heat!

Don Matéos, in his remarks on the sexes, chaste without being rigorous, is constantly warming our imagination without corrupting our heart. One of the Spanish physician's proposals is, that the law should forbid those scandalous marriages between decrepid age and blooming youth, which we every day witness! Don Matéos treats, as may be supposed, with caution on the subjects of incubus, succubus, and demonianism, which jugglers of every description, and even the highest dignitaries of the state, have not been ashamed to employ as the means of executing every infamous project—every execrable species of vengeance.

The importance of public hygiene is generally acknowledged; but its utility is not sufficiently appreciated. Governments have not duly estimated the value of medical philosophers; and it is to a neglect of their council, that our prisons, barracks, and hospitals have become, in turns, the most frightful depôts of pestilence, and too often the graves of their wretched inhabitants! The practice of interment in churches is an abomination; it is to burn an incense on the altars of the Almighty, the odour of which may spread infection like pestilential miasmata!" Don Matéos ranks himself among the anti-contagionists, and asserts that the dreadful fevers which devastated Madrid in 1803-4, presented the characters of inflammation; were rarely of a putrid nature; and required the constant operation of antiphlogistics; particularly blood-letting, which was of singular efficacy. The French critic doubts this; but we are inclined to think that the Spanish physician is correct. The *former* believes as firmly in the contagious creed of yellow fever, as a good catholic does in the truth of revelation. The *latter*, who saw the disease, and experienced it in his own person, is of a contrary opinion. We need not say to what party the most credit is due.

The picture which the Spaniard draws of a puplic hospital is frightfully melancholy to contemplate. "Figure to yourself a vast edifice, whose architecture is calculated to astonish by its majesty, or please by its elegance, but whose construction is devoid of every thing that can contribute to salubrity! The medical officers are never consulted in any part of the fabrication or internal œconomy of the institution. Under the appellation of HEALERS (guérisseurs) the physicians, badly paid, and little respected, sink into insignificance, and become as careless and negligent as the host of menial attendants. The miserable patient, who hopes for succour and consolation from his doctor, can scarcely address him when he is passed by without an answer! Charged with the office of visiting a great number of sick, the physician sees little or no sickness. The food and the medicines most slovenly prepared, are still more negligently distributed. The linen, and other furniture of the kind, are bad in quality, and not regularly changed. The atmosphere of the wards is impregnated with fœtid exhalations and no means of ventilation or purification are put in force to remedy the evil. In vain does the unhappy patient look around for some sensible being who may commisserate his sufferings—wherever he turns his eyes, the most doleful objects are presented to his view. Abandoned by their parents or friends, overwhelmed with chagrin and sickness, some are poisoned by the blunders of the stupid apothecary, while others, fall victims to the ignorance of the surgeon, who having made three or four unsuccessful attempts on the vein, in the fifth goes dash into the artery!

The French writer (Chaumeton) acknowledges that the Spaniard's descriptions will even apply to Paris. "Tout ce que dit le Medecin de Madrid, je le retrouve à Paris. Dan le capitale de la France comme dans celle de L'Espagne, le vice domine au point que la virtu est. Le Parisien comme L'Espagnol, remplit scrupuleusement des formalités pueriles, des pratiques ridicules, tandis qu'il vole impudemment les devoirs sacrés d' epoux, de pere, d' ami, de citoyen."

"When I cast my eyes, says Don Matéos, on those vast and majestic edifices, erected by misguided piety, fanaticism, or vanity:— When I see the temples of religion metamorphosed into sinks of debauchery and prostitution that realize the scenes of the ancient Bacchanals, I cannot help breathing a fervent prayer that these monuments of superstition were consecrated to the beneficent HYGEIA. The worthy ministers of this august Goddess would advantageously supersede those *pious* bands of idlers, whose greatest virtue is inutility."

Don Matéos concludes his work, by deploring the profound state of abjection into which the healing art is plunged in Spain. Will it be believed that the Universities, the Professors of Divinity, of Law; nay, even the Tutors themselves, would blush to be seen in company with the Professors of Medicine! The most petty artizan would be despised by his fraternity if he brought up his son as a Physician! The Government promotes, with all its influence, this strange perversion of ideas, this worse than Vandalism! It heaps humiliations on the heads of those benefactors of mankind; and seems to fear lest the learned Physician and Philanthropist should unmask sacrilegious abuses, and lay open those crimes which, though *authorized*, are not the less detestable.

Such are the sentiments of Don Matéos, whose manly expressions of indignation the French Editor has softened, but does not condemn. That the *rotten fabric* of such a government must soon totter and fall, needs hardly to be predicted in this age of spreading knowledge and unfolding faculties. That every base tyrant, superstitious monk, and cruel inquisitor, may be buried in the ruins, is the fervent prayer of the Medico-Chirurgical Journal.

---

### *To the Editors of the Medico-Chirurgical Journal & Review.*

——————Hic murus aheneus esto,
Nil conscire sibi, nulla pallescere culpa. Hor.

GENTLEMEN,

The peculiar views which Dr. Spurzheim entertains, with regard to the Anatomy and Physiology of the Brain and Nervous System, having engaged a considerable share of attention throughout Europe, the injurious treatment which he has met with in this city, instead of being a circumstance of local or temporary concern, will in all probability be, ere long, commented upon in other countries, so as to reflect ultimately much discredit on our national character for science as well as for hospitality. This weighty consideration, together with an innate love of justice, has prompted me to put forth a short outline of facts to the public; and I really do not know so fit a medium for such a statement, as the spirited and independent Journal of which you are the directors.

As I mean to state nothing, save that of which I am perfectly certain from my own personal knowledge, I shall confine myself to a history of Dr. Spurzheim's proceedings since his arrival in this city, which was about the end of last June. Previous to his coming hither, the scope of his investigations had been misrepresented, his anatomical views contradicted, and his physiological doctrines derided by an anonymous writer in the *Edinburgh Review* for June 1815, who, regardless of the decorum due to science, "*conscientiously*" mingled detraction with criticism, and traduced the moral character of him whose system he was endeavouring to impugn. The lovers of learning were grieved to find, that a review, whose pages are generally distinguished no less by profound views than by elegance of composition, should contain any thing so petulant, shallow, and dogmatical, as the article in question. If this critic be assailed by his own weapons, we shall find him by no means invulnerable. Throughout the whole article, we observe a constant substitution of contradiction for proof, sneering for argument, and ridicule for reasoning; and as for the style, it is studded with quaintness, colloquial idioms, and puny witticisms, all in the very spirit of bad taste. I cannot better sum up my opinion of this writer, than in the words which the uncourtly *Tacitus*, speaking of the faded eloquence of Ancient Rome, applied to one of the public declaimers of his time: "Omissâ modestiâ ac pudore verborum, ipsis etiam quibus utitur armis incompositus, et studio feriendi plerumque detectus, non pugnat sed rixatur."*

Dr. Spurzheim sank not under this cruelty of criticism, which he bore with a serenity of deportment worthy a man of science. On the contrary, his moral character appeared more bright in the eyes of those who knew him, simply from being contrasted with the foulness of the epithets that had been thrown upon it. He came to Edinburgh, therefore, not to indulge feelings of personal irritation, but in a spirit of meekness, anxious to find out his opponent, for no other purpose, than that he might convince him, by ocular demonstration, of that peculiar structure of the brain, which he had described in his works and plates.

I had the good fortune to be present at his first demonstration, which took place before a considerable number of eminent anatomists; the person also was there, whom rumour alleges to be the author of the offensive article in *the Review*. I marked the conduct of that individual, and if the outward deportment could be viewed as an indication of what was passing in the mind, he was certainly labouring under suppressed emotion; and more than once he tried to disembarrass himself by pulling from his pocket, and reading, or pretending to read, the superscription of a letter. He generally contented himself with distant hurried glances at what was demonstrated, and upon the whole, seemed both uneasy and inattentive.

I am the more minute as to those facts, because he has since alluded to this demonstration, as being by no means satisfactory; he was probably, however, the only individual to whom it was *not* satisfactory.

It is unpleasant to be obliged to comment thus on the conduct of any individual; and although in the present case, such a mode of proceeding is strictly retributive, I would rather have abstained from doing it, had not both the first and second demonstrations been lately mentioned *in print* by the individual in question; but after all, where is the unfairness? "*Quis tulerit Gracchos de seditione quererentes?*"—Surely, he who wrote the abovementioned article in the *Edinburgh Review*, ought to be the last to complain of unfair personalities.

On a subsequent occasion, Dr Spurzheim, with his usual readiness, demonstrated the brain to about two hundred spectators, among whom were several of the medical professors, and other competent judges. It was previously concerted, that his opponent should ask him questions; and by so doing, it was hoped, to give him and his doctrines a public and final overthrow. The

* De causis corruptæ eloquentiæ.—It would be well if some *Reviewers*, before proceeding to the execution of their office, would consult the Essay just referred to. It would, perhaps, teach them to distinguish true from fasle taste in composition.

scene was most interesting to the audience. Dr. Spurzheim, in his usual masterly manner, proceeded in the demonstration, and like the "admirable Crighton" sustained for upwards of *four hours and a half*, and in a language which was foreign to him, a public disputation with his adversary, explaining himself in terms at once philosophical and perspicuous, and very successfully and coolly ridding himself of the disengenuous cavilling about words with which it was sought to embarrass him. During this public disputation, it is pretty generally admitted, that there was, in one quarter, a right plentiful lack of temper, as well as of argument, and that had *Rare Ben Johnson* been alive to witness the scene, he might have found hints for improving one of his best characters, viz. that of the "Angry Boy" in the "Alchymist."

I am thus particular in my statement, because attempts have been made to misrepresent the feelings and judgement of the audience, which were unquestionably in favour of Dr. Spurzheim, in the proportion of at least twenty to one. It would have been too much to expect *perfect* unanimity on a question and an occasion of this sort; for little does he know of human nature, who is not aware what erroneous impressions on "the mind's eye" the *mirage* of prejudice and predilection now and then produces.

Since this notable occasion, Dr. Spurzheim has dissected the brain before the Royal Physical Society, and repeatedly to mixed audiences; and it is but bare truth to say, that I do not know any man of sense or candour who does not bow to the correctness of his pathological views, and admire the beautiful accuracy of his demonstrations. It has long been customary in anatomy for the names of individuals to be handed down to posterity, in connection with those parts of our frame which they were the first to discover, or accurately to describe; in this view, I will venture to allege, that Dr. Spurzheim has done more to deserve the meed of this sort of immortality than perhaps any living anatomist, whatever be his name or fame.

Had Dr. Spurzheim's persecution ceased here, I should not, in all probability, have troubled you with this statement; but within these few months an expensive pamphlet has appeared, the cumbrous materials of which have been raked out of old anatomical books, for the obvious purpose of depriving this meritorious gentleman of the rank he holds as a discoverer, and of implicating his moral character for candour and good faith. At page 43 of this pamphlet, the writer is pleased to aver, that the spectators of Dr. Spurzheim's demonstrations were incompetent to judge of what was shewn them. This proves that the author's modesty is on a level with his other powers. The passage, however, comes in very happily to enliven the dryness of his other matter, for doubtless, it is one which must relax the frown of criticism, and sheath all its acrimony in irrepressible laughter.

I would be strongly tempted to enter at large into the demerits of this performance, had I not already occupied so large a space of your Journal; this being the case, I must be content to conclude with two or three general reflections.

It must be admitted by all, that our knowledge of the physiology and pathology of the brain is yet in its very infancy. Why then persecute Dr. Spurzheim for attempting to elucidate this science? Until his investigations, no advance had been made in this department of knowledge for many years; because the despair of medical philosophers has set it aside amongst the arcana of Nature. Any step forward must therefore be an advantage; for, as Mr. Burke has well observed, "Science is never more apt to become corrupt than when it is allowed to stagnate."

I am the last that would propose to bridle discussions in matters of science; on the contrary, let it be free as the winds of heaven. But every man of feeling would surely wish to draw with me, a line of distinction between legitimate, temperate criticism, and coarse *brochures*, undertaken in malevolence, and executed in imperfect mechanical views of the subject agitated.

In the above observations I have adhered rigidly to the truth. I trust I have as high a sense of the "nil nisi verum" as any man breathing; and besides, respect for your independent Journal would not permit me to make it the vehicle of wilful falsehood.

I conclude by saying, that I have no connection with Dr. Spurzheim, and that he knows nothing of my having taken this step. To my respect, as to that of every other man, he is entitled by his superior talents, as well as his excellent and amiable character; but upon me he has no peculiar claim, save that which I am ever ready to allow to injured merit. I am, &c.

VERIDICUS.

*Edinburgh*, 1817.

Anonymous strictures on anonymous writings are fair. But Veridicus is known to us; and his character is beyond suspicion of misrepresentation. In these islands we hope and trust that the STRANGER's cause will never want an advocate. While the MEDICO-CHIRURGICAL JOURNAL exists, the advocate of injured merit shall never want a medium of conveying his sentiments to the public ear.

Hanc veniam petimus damusque vicissim. EDITORS.

---

### *Influence of the Climate of India on the Human Constitution.*

Although it is an invariable maxim, that the MEDICO-CHIRURGICAL JOURNAL never copies from any *Medical Journal* published in Great Britain; yet, it is not hereby restrained from selecting professional intelligence from works not strictly medical. Under this impression, we shall present our readers with a concise *analytical sketch* of an article lately published in the JOURNAL of SCIENCE and the ARTS, by Dr. Scott, late of Bombay, on the Arts of India.

Dr. Scott was long a resident in the climate of which he speaks, and has strongly felt its influence in his own person; his observations are therefore entitled to respect. We shall touch first on the *immunities* which the Doctor supposes a tropical climate to confer on the constitution of man. 1st. CANCER is nearly unknown between the tropics. During twenty-five years, Dr. Scott only saw one case of it in a person who brought the rudiments of it from Europe. 2. Phthisis Pulmonalis is not common, though it does sometimes occur. The lungs, however, suffer requently from abscess, and other affections of the liver; and in these cases, it is no easy matter to distinguish such complaints from true phthisis pulmonalis. 3. Scrofula is rare, though particular causes sometimes call it into action. 4. Dr. Scott never knew an instance of biliary calculi between the tropics. 5. The formation of stone in the urinary bladder is nearly unknown in hot climates. 6. Gout is rare between the tropics, as is acute rheumatism. These observations must be taken as limited to a part of India, viz. between Bombay and Ceylon; for had the Doctor visited other tropical climates, he would have known that rheumatism, both acute and chronic, is by no means rare.

We now reverse the picture. While those glands that are the seat of scrofula are less generally diseased in India than here, other glands suffer more in tropical than in northern climates. We need not say that the liver and spleen are the organs alluded to. " I have *fancied*, says the Doctor, that I could see *mechanical* causes for some of the derangements of the liver in a hot climate. The resinous part of the bile seems to be there more abundant. It appears occasionally to separate from its union with soda, when it stagnates in the liver and enlarges it, giving rise to all the phænomena of chronic hepatitis." We cannot admire the *mechanism* of this ingenious hypothesis, and are well convinced of its *crazy* nature. There is more solidity in the following passage:

" The calces of mercury do certainly give the utmost relief, both in acute and chronic hepatitis. While in the acute kind we employ between the tropics the antiphlogistic plan, blistering, blood-letting, and especially purgatives, we ought not for a moment, if the disease is severe, to delay the use of the calces of mercury internally, with the ointment externally, as being of more consequence than all the other means in our power. No condition, to which human nature is exposed, is more deplorable than that where an abscess has taken place in the liver. I know of no sufficient security in that climate against such an evil but mercury. As soon as the mouth gets sufficiently affected, and the system is impregnated with it to a proper degree, the pain, the fever, and the distress abate, and the patient remains quite secure from the risk of abscess, provided we have not used the remedy too late; and when such a change has taken place as must necessarily end in abscess." P. 201.

Dr. Scott, anxious to find out a substitute for mercury, that might be less injurious and equally efficacious, thinks that he has not been quite unsuccessful. This substitute is the *nitric acid,* both internally and externally applied. It was rather *nitro-muriatic acid,* however, than the pure acid which the Doctor used. The anti-syphilitic powers of this remedy, which Dr. Scott many years ago insisted on, are now, it appears, dwindled down to *anti-syphiloid,* and this circumstance may explain the reputation which nitric acid obtained in England, some years since, in (supposed) venereal affections. The truth is, that every case reported as cured by this remedy was one of pseudo-syphilis! Speaking of syphiloid diseases, Dr. Scott observes, " This *state of syphilis* is thought by some able and eminent men to be a *new disease,* and arising rather from the *consequences of the remedy* than from the poison of syphilis." Surely there is here some confusion—some inaccuracy. If it be a *new disease,* it cannot properly be called a *state* of the old; and if it be merely " the *consequences of the remedy,*" it cannot be a new disease; for these consequences must have arisen at all periods since the remedy was first found out. We believe the opinion of the best informed to be this, that pseudo-syphilis is a disease essentially different, in its nature and treatment, from syphilis, but frequently simulating the forms of the latter so closely, that experiment (not experience) alone developes the distinction. Again, we do not think " that our able and eminent" men consider pseudo-syphilis as the " consequences of the remedy;" but that it is very generally aggravated by mercury, especially when pushed too far, or continued too long.

Dr. Scott is of opinion, that in it [pseudo-syphilis] the poison of syphilis still exists, remaining occasionally dormant, and becoming, from unknown causes, active and injurious, and exerting again all its specific effects. " I think (says he) that the *cause* of pseudo-syphilis is a scrofulous habit, acted upon *at once* by the poison of mercury and the poison of syphilis." But if the poison of *real* syphilis be present in pseudo-syphilis, how is it that many cases—nay, most of the *latter,* get well without mercury at all? Again, if the action of the mercurial and syphilitic poisons on scrofulous diathesis constitute pseudo-syphilis, how is it that we every day see the disease *anterior* to the exhibition of mercury?—If Dr. Scott can unravel this intricacy, we shall feel much obliged to him. For our own parts, we see no possible means of his doing so.

Dr. Scott now earnestly recommends the nitro-muriatic acid bath [three parts of nitric and one of muriatic acid, diluted so as to taste about as sour as vinegar] in cases of pseudo-syphilis. Like the calces of mercury, this bath affects the gums and salivary glands, occasionally producing plentiful ptyalism, but never that nauseous smell, nor those fœtid ulcerations arising from mercury. When this effect results, it is prudent to omit the medicine, or expose a smaller surface of absorption. If continued too long, a degree of restlessness is superinduced. In this country, it will be sufficient to bathe the feet and legs once or twice daily, for twenty minutes or half an hour at a time. Dr. Scott considers the trial either internally or externally as incomplete, unless some degree of ptyalism, or evident constitutional effect, result from the acid. Dr. S. asserts, that he has seen "recent chancre, and that of a bad kind," completely removed in about a week by the nitro-muriatic bath. Much do we suspect that these "chancres" were *syphiloid* sores: we could wish to have a description of their appearances.

Dr. Scott gave the bath a trial, in India, in various other complaints; and, in many instances, with success. In some affections of the skin it was useful, but might not this be from the simple ablution? In constitutions broken down by fever or long-continued disease, the nitric acid bath had a tendency to renovate; and our author has seen the happiest effects from it in aphthæ of the mouth and intestinal canal. In chronic hepatitis and a bilious disposition, he has used it with much advantage. In many female complaints, and in men worn out with obstinate intermittents, it was found beneficial. In short, and as a general rule, Dr. S. found the acid bath advantageous and salutary in all cases where mercury is useful, and with the additional advantage that the acid treatment is attended by neither injury nor inconvenience. Since Dr. S. came to London, he has seen numerous trials made by different medical practitioners with this bath in scrofulous diseases. Some of the cases were of long standing, and of the worst kind. The result has been, upon the whole, favourable; though none of the patients had, at the time of his writing, used it three months. In almost all, the health was improved, and some of the sores had healed, or shewn tendency to heal. He has seen remarkable relief in several cases where the neck was much affected, much swollen, and with many glands in a state of suppuration, so as to make the least motion painful and nearly impossible. In one girl, in particular, who was reduced to a dying state, by merely drinking the acid her health and strength have greatly improved, and the sores have healed or are healing.

Bearing great respect for the author of this paper, and anxious to diffuse the knowledge of any thing that can check the ravages of pseudo-syphilis and scrofula, we lay these particulars before our readers with more hope than expectation, that Dr. Scott's remedy will prove as efficacious in the hands of others, as it appears to have done in those of its proposer.

---

### *Mr. Stevenson's Oration before the Medical Society of London.*

On the 8th of March, the day on which the Institution of the Medical Society was commemorated, Mr. Stevenson (in consequence of the premature death of Mr. Royston, who had been elected Orator at the previous anniversary meeting) delivered an Oration, which commenced with some judicious reflections upon the necessity, the value, and the importance of the medical profession.

As the origin and progress of medical science had been frequently and ably treated of (by those who had preceded Mr. S. in this situation) from the remotest ages to the present enlightened period, the Orator confined himself to a brief outline of this, the first part of his subject. "Were our bodies so framed (he observed) as to be not only free from original defect of structure, but capable also of performing their several functions with undeviating regularity, we should, it is manifest, enjoy a state of perpetual health, and with equal moral perfection, we might fancy the reality of the fabled and visionary description of the golden age. The absence of that natural evil, which infuses into the cup of human felicity a bitter mixture, would in that event apparently have rendered existence an uninterrupted scene of pleasure. But this admirable piece of workmanship, the human frame, like every other part of creation, is destined to decay; not a moment elapses without a change, either natural or moral, sensible or insensible, having been introduced. The constituent parts of our wonderfully organized frame are unceasingly undergoing some modifications or alterations in their arrangement, and the whole is so delicately and exquisitely formed, as to excite much greater wonder, that we can *ever* enjoy perfect health, than that it should be highly susceptible of injury from external causes; that it should frequently fall into disorder, and, by incessant friction and activity, at length be worn out, like all other substances; being gradually decomposed into that primæval dust, from which, by the fiat of the Divine Artificer, it at first sprung into life."

The learned Orator then went on to point out the cause by which man would necessarily become affected with disease; dwelling, with great ability, upon the complicated structure of the human body, the effects of atmospheric changes upon it, its liability to accident, the consequences of long abstinence, repletion, improper regimen, immoderate or insufficient corporeal exertions, &c. Exposed to wants, to external dangers, and internal weakness, Mr. S. remarked, the impatient mind of man would soon apply itself to the invention of means calculated to lessen, if not to obviate their effects. The personal feelings of the sufferer, and the anxious solicitude of his friends, would naturally excite a spirit of industry and research to procure alleviation. The rude and uncultivated mind, ever prone to indulge in the delusive hopes of superstition, resorted, upon such emergencies, to charms and incantations, which, however absurd and insignificant in themselves, doubtless, in some instances, by their power over the imagination, and by the confidence which they inspired, would be found salutary and efficacious. By the experience obtained from actual observation, what appeared to do harm would be studiously avoided, whilst those means were resorted to which had been found to prove beneficial.

After tracing the rise and progress of the medical art among the Babylonians and Egyptians, Mr. S. proceeded to shew the high rank it maintained among the Grecians, by whom it was cultivated with a zeal proportionate to its dignity and importance. From this, the Orator proceeded to remark on the utility of the profession, the difficulties connected with its honourable exercise, and the best modes to ensure its respectability and advancement. Few arguments are necessary to prove, that those who employ themselves in administering to the preservation of health and the removal of pain and disease, and consequently the prolongation of life, ought to be regarded as amongst the greatest benefactors of their species; but it was well observed, that, "by the proper exercise of this God-like art, we possess the enviable power of doing good to our fellow creatures, in a way the most necessary to their wants, and most interesting to their feelings. By this we are enabled to confer that which sick kings, if cut off from the source of relief, would not hesitate to purchase at the price of their crowns; that which the most exhaustless wealth alone cannot command, nor state nor rank bestow. We shall be able, by the diligent attainment and exercise of medical knowledge, to alleviate and remove disease, the most distressing and the most insupportable of human afflictions, and to substitute the blessing of health, "which he who possesses has little more to wish for, and he who is so unhappy as to want, wants every thing with it. Hence physicians, properly qualified, and duly fulfilling the duties of their important vocation, have, in most civilized countries, been held in the highest veneration and respect, and in some foreign states have been advanced to lofty civic honours."

The profession of physic, Mr. S. remarked, although not possessing so many allurements as those of divinity and law, is by no means barren of attraction. It opens the way to reputation and wealth, and raises the successful practitioner to a level, in the intercourses of common life, with the highest classes of society. It must be acknowledged, however, that the exercise of the medical profession is not attended with the most pleasing intercourse. It views human nature in the most disagreeable, and too often fretful moments, when it is almost impossible to please, or to avoid even offence, by its best services: so much foundation is there for Dr. Johnson's very striking picture of it, viz. "that it is a melancholy attendance on misery, a mean submission to peevishness, and a continual interruption to pleasure."

The requisite qualifications to enable a physician to practise the arduous duties of his vocation with advantage were next detailed. The communication of medical knowledge, through the medium of the discussions of the Society, were ardently recommended, and an eloquent tribute paid to the memory of the deceased President, Dr. Lettsom.

The Orator then observed on the defects of the earliest state of the medical art, and ventured some suggestions on the succeeding and on the present practice of the profession, admitting all due importance to the writings of Hippocrates, Galen, Aræteus, and Celsus, yet contending for the great superiority of the moderns over the ancients, both in the description of disease, and for acute and accurate observation. The importance of attending to morbid anatomy was duly insisted on. After remarking on a few of the errors that have at different times prevailed, from the introduction of ill-founded theories, Mr. S. justly observed, that "it is hard to say what is the prevailing theory of disease." Many morbid phænomena have been referred to the mysterious agency of electricity and Galvanism. Our attention is at present more particularly directed to the functions of the digestive organs; and of these, the liver has had the imputation of giving birth, like Pandora's box, to an almost innumerable description of evils. The bile, in the character of excess or defect in quantity, or vitiation in quality, is reproached with being the prolific parent of a dreadful catalogue of nervous, dyspeptic, arthritic, and various other complaints, of the most discordant nature and Protean form. The due and healthy action of this viscus, however, the Orator admitted to be of the greatest importance in the œconomy.

Our limits will not admit of our following the learned Orator through his ingenious view of this subject; we must, therefore, hasten to conclude, by noticing a few remarks made by Mr. S. respecting Amaurosis. This disease is universally admitted to consist in an impaired or total loss of sensibility of the nerve appropriated to the sense of vision, the tunics and humours of the organ retaining their natural transparency. Whatever causes are capable of deranging the function of the optic nerve, by acting directly upon its origin in the brain, or its expanded termination in the retina, or, lastly, by its sympathy with some remote viscus, as the stomach, may (said Mr. S.) become the exciting cause of this complaint. Now as these are various, so must the modes of treatment be equally diversified. Hence it follows, that to attempt the cure, in all instances, by the same means, as is too commonly done by stimulants and nervines, on the supposition that the disease arises from a weakness or torpor of the nerve, must be no less irrational than unavailing. During the last few months, Mr. S. had met with several cases of this disease, characterized by so great an obliteration of sight, that the patient was able to distinguish only the outlines of objects or luminous rays from total darkness. The pupil was fully and immovably dilated, without any appearance of fulness of the vessels of the conjunctiva, or more than a sensation of very slight tensive uneasiness in the globe of the eye. The disease commenced, in all the instances, very suddenly, chiefly in the night, and was not preceded by any of the usual precursory symptoms. The subjects were both male and female, of various ages and stations in life. In some cases one, in others both eyes were equally affected, without any preceding or accompanying indisposition. After very careful and minute inquiry, Mr. S. succeeded in tracing the origin of the attack to exposure of the eye to the partial application of cold air, when the body was preternaturally heated.

With regard to the pathology of the disease, time would not allow him to enter into particulars, which, however, we hope he will be shortly induced to lay before the public. He seemed disposed to suspect, that the blindness in these cases arose from the sudden repulsion of blood from the superficial to the deep-seated vessels of the eye, in which perhaps there might previously exist a *partial* plethora. The defect, therefore, of vision, in these instances, is probably the consequence, not of an indefinable and inexplicable torpor, but of a direct compression of the fibrous substance of the optic nerve, by the casual enlargement of its central artery, or of its medullary expansion in the retina, by the blood-vessels, so abundantly distributed through its pulpy texture, becoming, from the above cause, incidentally and preternaturally over distended. Without contending for the accuracy of the theory suggested, Mr. S. had the satisfaction to add, what is of much greater importance, that by means of active general and topical depletion, each of his patients, in the several cases to which he alluded, obtained the speedy and complete restoration of sight.

The Oration was very numerously and respectably attended; and there is reason to regret, that the learned Orator could not be prevailed upon to print it, it being a production equally creditable to the Society and its ingenious Author.

---

*The following very interesting Trial is well calculated to give food for reflection to the Juridical Physician. We shall state it, unaccompanied by comment.*

### CORNWALL ASSIZES.—LAUNCESTON, March 31, 1817.

### CHARGE OF MURDER.

The following interesting trial for Murder occupied the Court this day from eight in the morning until near nine in the evening. The number of persons anxious to be present was so great, that not a single bed could be procured in Launceston for several days past; and the croud at the doors of the Court this morning was so excessive and violent, that the additional precaution of bars and double bolts were found insufficient. The javelin men were overpowered, and the High Sheriff himself, in his full court dress, was several times compelled to descend to the ignominious office of door-keeper.

The prisoner, Robert Saul Donnall, is a young man, aged 26, and carried on the business of a Surgeon and Apothecary at Falmouth. In July last he married the daughter of Mrs. Elizabeth Downing, of that place, widow; and he now stood charged with the wilful murder of his mother-in-law, on the 3d of November last, by administering arsenic in a cup of cocoa, upon bread and butter, or in some other undiscovered manner. His wife is upon the point of being confined.

The prisoner being put to the bar, was arraigned, and pleaded Not guilty; after which Mr. Serjeant Lens stated the facts to the Jury in a very candid and temperate address: they are all contained in the depositions of the following witnesses.

Mr. Gilbert Abrahams swore, that he lived at Falmouth, and that in February, 1816, the prisoner applied to him for loans of eleven, fourteen, and fifty pounds; stating, that he was in considerable distress for the money, but that he should soon be married to Miss Downing, daughter of the deceased, when the whole should be repaid. On the 23d of May he applied for fifty pounds more, observing, that it would either be his making or his ruin. He was married to Miss Harriet Downing in July last, and the witness several times applied for the repayment of the loans. About three weeks before the death of Mrs. Downing, he said that his mother-in-law would not live long, and that then the witness should have his money.

Mr. Samuel Downing, third son of the deceased, deposed, that on the 19th of October last, Mrs. Downing, the mother, went to drink tea with the prisoner, her son-in-law; the witness had previously dined with her, and she appeared in good health and spirits. She returned home after supper, and, about twelve o'clock, complained of great sickness at her stomach. She vomited, and was attacked by a violent cramp in her legs. The prisoner attended her, and she was better on the following day; but she did not entirely recover for more than a week. On the 31st of October she drank tea at the house of her son John. On Sunday the 3d of November, she was again invited to tea by the prisoner, to meet her daughter and another son-in-law a Mr. Jordan. On this occasion she observed, "You know the last time I was at your house I was taken ill; I hope it will not be so again." The witness dined with her, and she ate heartily. He went to the prisoner's at about half past five, when he found Mrs. Donnall at the table, on which were the urn and tea things, and a plate of bread and butter. The witness and the prisoner sat on the same side of the fire, while Mrs. Downing and her son Edward sat together on the opposite side. The party was only waiting for the cocoa, which was brought up soon afterwards by the maid servant. When it was poured out, the prisoner left his seat, and went to the table for the purpose of handing the cups and bread and butter. The attention of the witness was attracted by seeing the prisoner going round, and passing behind the chairs of some of the party, with the plate in his hand, towards Mrs. Downing; in doing which, he spilt a part of her son Edward's tea upon her gown. Edward exclaimed, "Donnall, what are you about?" The prisoner made no answer, but offered the bread and butter to Mrs. Downing, who took a piece of it. The prisoner then returned to his seat. Just afterwards the servant boy called the prisoner out of the room. There was nothing unusual in the prisoner's handing the bread and butter or tea; for, when the servant did not attend, it was his practice to do so. The witness did not see who handed the cup of cocoa to his mother, but he saw her drinking it. She took a second cup, and while drinking it she complained of being sick, and in a few minutes returned home with her son Edward. Her expression was, that she felt unwell, and withdrew from the fire. While she was in the act of returning, the prisoner came again into the room. Immediately she got home, (the two houses joined) she desired that a basin should be brought, and she vomited ; observing, that she hoped she should not be so sick as she had been the last time she came from Donnall's. The prisoner and his wife entered soon afterwards, and the deceased was prevailed upon to go up stairs to lie down on the bed : she complained of great heat. The witness then went out, and returned about nine o'clock : he found in his mother's house the prisoner, William Downing, and Mr. Jordan. In the course of the evening, the prisoner frequently went up stairs to Mrs. D. bringing word only that she was still sick. The chief topic of conversation down stairs was field sports, and the prisoner said nothing about administering any medicines. The witness went to bed about two o'clock, having recommended an emetic an hour before ; but the prisoner replied, that gentle means must be used, and that he had given Mrs. Downing something to compose her. When the witness was going to bed, he asked the prisoner whether he should stay longer ; and the answer was, that he should wait half an hour, to ascertain the effect of the last pill. The witness entered his mother's room as he passed the door, and the prisoner went with him : she complained of violent pains in her stomach, and of cramp in her legs ; in order to relieve which, she wished to leave her bed. The witness then went to bed, and about four o'clock he was awakened by Mrs. Jordan, who said that her mother was much worse ; that Dr. Edwards had been sent for, and was then in the house. The witness dressed himself, and met the prisoner at the outside of the door of the deceased's room. Being asked why he had called in Dr. Edwards, the prisoner said, that about three o'clock he became alarmed by the failing of Mrs. D's pulse. The witness found her in a slumbering state, which, the prisoner observed, was a farther cause of fear for her safety. The deceased died about eight o'clock on Monday morning. The prisoner then left the house, and the witness did not see him again till eleven o'clock, when he remarked, that the body would probably swell ; that a shell should be prepared, and that it should be buried without delay : he had already observed a discharge at the nostrils, and the body could not be kept longer than Wednesday. The witness and his brother William were surprised at this intelligence. On Tuesday morning the prisoner came into Mrs. Downing's house, and said, that as he was going to Helsford or Monackan, to visit some patients, it would be as well for him to call at the church-yard at Mawnan, only about a mile out of his way, to see how the workmen went on with the vault ; but the witness said, that he did not think it was necessary to hasten them. Helsford and Monackan are about six miles from Falmouth, on the other side of the river ; and unless the ferry be crossed by the boat, the distance round is more than thirty miles. The prisoner, however, went ; and returning about four in the afternoon, he stated, that he had been at Helsford, and that in his way he had called at Mawnan, and hastened the workmen. On Wednesday the prisoner did not leave Falmouth, and on Thursday he came to the house of the deceased, accompanied by Mr. Jordan. On the previous evening the witness had received from Mr. Bull, the mayor of Falmouth, an anonymous letter, of which the following is a copy.

"Sir,—I shall, without apology, demand your most serious attention to a circumstance which has excited considerable notice in this town; and which, from the fact I shall mention, makes a minute and immediate investigation necessary. Mrs. Downing drank tea with her son-in law on Sunday evening, and returned home ill: after violent retchings and pains in her stomach, it soon ended in death. Something of the same kind she experienced before, when she drank tea at the same place. You will, I am sure, notice the similarity of her complaints to those of persons who have swallowed some corrosive substance; and the symptoms were the same, though not so violent, when she drank tea with her son-in-law before."

This letter was addressed to the Mayor, and the copy he had received the witness put into the hands of the prisoner in the presence of Mr. Jordan: while the prisoner was reading it his hand trembled violently; and before he had finished it, it dropped from his hand on the floor: he said "It is a villainous thing, and has been done on purpose to ruin me in the town, and my practice will be broken up in consequence." At about two o'clock on the same day the body was opened: the witness remained down stairs, and the prisoner brought word that the heart and liver were found perfectly sound, though there was an appearance of a little inflammation in the stomach. The deceased was buried on the Saturday following, and the funeral was attended by the prisoner. The Coroner's Jury continued to sit after the funeral; and just before the witness's brother Edward was called before it, the prisoner observed, "I was not present when the first cup of cocoa was handed. Edward you know I was not present. You had better say you gave your mother the first cup." They had been previously talking on the subject, none of the party being able to remember who handed the first cup. Edward admitted that he gave the second, and left the house without answering the observation of the prisoner The next morning the prisoner called upon the witness to speak of the long examination he had undergone at the Town Hall; saying, that the witness and Mrs. Jordan would probably be called upon: the witness observed, that the verdict had been already returned, that Mrs. Downing died by poison; at which the prisoner did not seem surprized, but merely observed that it was a mistake.

When cross-examined by Mr. Serjeant Pell, (who led on behalf of the prisoner), the witness said, that he was not present at the Town Hall when his brother Edward was examined. He did not know of the anonymous letter until Wednesday night. The prisoner was getting into good business in Falmouth: the witness and he had always been most intimate friends, and had never had any dispute. The deceased was 64 years old; and on Sunday the 3d November, ate rabbit smothered with onions for dinner, and drank beer. The room where the party drank tea was small and oblong. When the prisoner returned to the company, after he had been called away, he said that he had been to draw a tooth. The witness did not know what became of the bread and butter in the plate after his mother had taken a piece. He had no reason to look for an anonymous letter; and when he gave it to the prisoner he had no suspicion of him. When the prisoner said it was done to ruin him in his business, the witness observed, that he believed it to be a malicious thing. The deceased had been in the habit of drinking cocoa latterly. Dr. Edwards was called in by the prisoner.

Mr. Edward Downing swore, that he gave his mother her second cup on the 3d of November; but he did not know from whom she received her first. The prisoner had told him, that he had better inform the Coroner's Jury that he (the witness) had handed the first cup; to which the witness made no answer. The prisoner had previously remarked, that Mrs. Downing had £600 a year.

Cross-examined.—His mother had asked him to come and occupy the chair next to her, because her son Samuel was entering the room. He saw the prisoner hand the bread and butter to the deceased. The witness had never admitted that he handed the first cup to Mrs. Downing. Mrs. Donnall poured out both the first and second cups, but how much cocoa she took herself the witness did not know: she drank cocoa. When the servant did not wait, the prisoner was in the habit of handing the tea, &c. The witness did not know who wrote the anonymous letter.

Re-examined.—Mr. Bull, the Mayor of Falmouth, had asked the witness if he had not handed the first cup of cocoa, but he had denied it; the same question was put by Mr. Richards, who attended the Coroner's Jury on behalf of the prisoner, and the same answer was given. When the prisoner handed the bread and butter to the deceased, he did not go across the room the shortest way but he went round behind some of the chairs.

Dr. Richard Edwards gave evidence, that he had been 16 years in the profession. When called in between four and five o'clock on the morning of the 4th of November, he asked the prisoner some questions respecting Mrs. Downing: the prisoner said, that she had an attack of the *cholera morbus;* and that she had been similarly affected about a fortnight before. The witness went into her room, and found that she required a little rousing before she could answer any questions: she complained of great heat, and her pulse was frequent and fluttering; she had no sickness at that time, but was in very great danger. He understood from the prisoner, that he had given her an opening medicine, an emetic, a saline draught, and some opium. The witness wrote a prescription, with a view to remove something offensive which appeared to be in her stomach or bowels. He had never met with a case of

*cholera morbus* that had produced death in a shorter time than three or four days; and he was of opinion, that that disorder was not the cause of the death of Mrs. Downing. On Thursday, the 7th of November, the witness was requested to go and examine the body, by opening it, having previously heard of the suspicion of poison. He saw the prisoner at the house, and a surgeon of the name of John Street. After taking the body out of the shell, the witness perceived that the prisoner was tucking up his sleeves and preparing to open the body, but the witness told him that he must have nothing to do with the operation. On opening the stomach, the contents were poured into a bason, and after the lapse of a few minutes they were examined, but no deposit of any thing heavy or solid could be discovered. The stomach was in a state of stellated inflammation, and both the coats of the stomach were softened, as if by the action of some corrosive substance; one of them could be scraped off by the finger nail. The blood vessels of the stomach were more than ordinarily turgid, but the liver and lungs were sound: the heart the witness did not examine. The bason with the contents of the stomach had been carefully placed on a chair; and the witness observed at the time aloud, that they must be preserved, and the prisoner must have heard it. They then proceeded to inspect the intestines, which were also inflamed, and the witness felt certain that it could not have been produced otherwise than by the operation of an active poison. After this investigation, the witness turned round to the bason, and was surprised to find it empty: he asked the prisoner what he had done with the contents of the stomach, and he replied that he had thrown them into the chamber vase, where was a quantity of water, with which the intestines had been washed. The witness said, that it would give him a great deal more trouble in making experiments to ascertain if any poison were in the stomach, as he must evaporate about two quarts of water. Mr. Street took charge of the chamber-vase and its contents, and it was pushed under the bed. The witness afterwards applied to it various chemical tests, and they indicated the presence of arsenic in a fluid, but he could not detect it in a solid state. He had not the slightest doubt that arsenic was the cause of the death of Mrs. Downing, and he should have been of that opinion, had he not analyzed the contents of the stomach.

Cross-examined. — He said, that *cholera morbus* was produced generally in hot seasons by the overflowing of bile, which irritated the stomach; it was a very painful and distressing complaint: cramp was frequently occasioned by a bilious attack; laudanum was sometimes given in cases of *cholera morbus*, but not in large quantities. The tests he had used to detect the arsenic were, first, sulphate of copper, with a small quantity of carbonate of potash: and when applied to the contents of the stomach of the deceased, the precipitate was green. Next he had used nitrate of silver in the same way, and the precipitate was yellow. Both these appearances showed that arsenic was present. He had tried bile by the ame test, and onions (having been told that the deceased had eaten of them); but it had exhibited no appearance like that of arsenic. He had not attempted to reproduce the arsenic, not having a sufficient quantity; but he was quite satisfied with the former experiments. The witness did not recollect that the prisoner had required any other test to be applied; but he had sent for a part of the contents of the stomach after all had been used.

Mr. John Street, who had been called in by the prisoner, but who had ceased to practice for five years, agreed with Dr. Edwards in his account of the appearance of the stomach, &c. of the deceased.

Dr. Edwards, on being recalled by Mr. Justice Abbott, said, that eighty parts of cold water would dissolve one part of arsenic; that warm water would dissolve a larger proportion, and that then two or three tea-spoons full, or a table-spoon full would probably produce death.

Mr. S. P. Street confirmed, in one or two particnlars, the statement of Dr. Edwards.

Mr. W. Downing, in his evidence, detailed most of the circumstances related by his brother. The witness examined the body, and found it was not true that there was any running at the nostrils.—When the opening of the body was spoken of as necessary, the prisoner said that he should not have time to attend, as he must go into the country on business. After the corpse had been examined, the witness was in a room with the door open, and hearing a pouring of liquid from one vessel into another, he looked out, and saw the prisoner passing out of the apartment where the operation had been performed: he afterwards returned to the room, and the witness again heard a noise like the pouring of water. Cross-examined.—He said that he did not go into the room to see by what the noise had been occasioned.

Dr. Edwards, being again recalled by Mr. Justice Abbot, said, that the body of the deceased did not swell before the interment, nor was there any running at the nostrils, or other symptom of putrefaction: he saw nothing to warrant a medical man in stating, that a shell was immediately necessary, and that the body must be interred on the next day but one after the death. The witness was not competent to state, whether putrefaction commenced soon after death by the *cholera morbus*.

The case for the prosecution being here closed, the Learned Judge called upon the prisoner for his defence: who read a paper, shortly denying the charge, and expressing his confident reliance on the impartiality of the Court and Jury.

The following evidence was then adduced by his counsel, Mr. Serjeant Pell and Mr. Gifford.

Mary Coombe swore, that she was servant to the prisoner in November last; she had previously lived with his father and mother. Mrs. Donnall was unwell, and took cocoa as well as Mrs. Downing: the cocoa was generally kept in the kitchen, and on the

3d of November it was made by the witness; there was about a pint and a half of it. The witness took away the tea-things after Mrs. Downing went; some cocoa was left in the pot, which was warmed again for Mrs. Donnall's breakfast next morning.—Mrs. Downing also left half her cup full, which the witness drank as soon as she had brought it into the kitchen, and found no ill effects from it.

Dr. Neale, a physician at Exeter, deposed that he had frequently attended cases of *cholera morbus*, which was occasioned by a collection of putrid bile in the intestines: the effect of it was vomiting and purging, pain in the stomach, and cramp in the legs; it might be considered the most acute disease known in Great Britain: it ran its course most rapidly, and he had often known it kill the patient in twenty-four hours; and, if neglected, or improperly treated, in a much shorter time. The medicine he should prescribe, would be large doses of opium and a quantity of warm broth or water. The witness had heard the description of the state of the body of the deceased, given by Dr. Edwards, and he attributed the stellated inflammation, and other appearances, to nothing but the *cholera morbus*, though they did not indicate that disorder exclusively. The witness had frequently made experiments in chemistry, and he considered the tests that had satisfied Dr. Edwards of the presence of arsenic in solution by no means infallible; the same green precipitate might be otherwise produced than by the presence of arsenic; the other test of the nitrate of silver was equally delusive, for the same yellow precipitate might be shewn if the nitrate of silver, or lunar caustic, were immersed in phosphate of soda. The only perfect and indubitable test was the reproduction of the arsenic by sublimation, by submitting the arsenic to the action of heat. He had tried experiments with a decoction of onions within the last five days, and applying it to the test of Dr. Edwards, an opaline cloud was formed; the addition of phosphate of soda completed the yellow precipitate. The green precipitate mentioned by Dr. Edwards, the witness had also obtained from a decoction of onions, by means of the sulphate of copper and phosphate of soda.

Cross-examined.—He had never opened the body of a person who had died of *cholera morbus*, but the appearance would be such as had been described.—Eating onions would produce the same effect as were seen in the decoction he had made. Phosphoric acid existed in all the fluids of the human frame; but whether more in the bile than in others, he could not state; but this with the onions eaten by the deceased, might produce the precipitate mentioned by Dr. Edwards.

Dr. Daniel, also a physician at Exeter, had attended several persons attacked by *cholera morbus*: the symptoms were precisely those felt by Mrs. Downing, and the remedy was opium.

Cross-examined.—He admitted that those symptoms might be produced by arsenic; he had never known any case of *cholera mor-*

*bus* so suddenly fatal, but he had known one nearly fatal in fourteen hours: the attack was sometimes rather sudden.

Mr. Tucker, a surgeon, deposed that the inflammation spoken of by Dr. Edwards, might have been occasioned by *hernia*, *cholera morbus*, and unascertainable causes. The prescription of Dr. Edwards was precisely the reverse of what the witness would have recommended; it excited, instead of allaying irritation. The witness had never opened any body after death by *cholera morbus*, but inflammation would, undoubtedly, be visible; he had made experiments with tests; he contended that those of Dr. Edwards were not infallible; on the contrary, the witness had tried them upon a decoction of onions, and found both the results of the green and yellow precipitates.

Dr. Cookworthy, practising at Exeter, said, that he was borne out by men of science in all countries in asserting, that the only infallible proof of the presence of arsenic, in a fluid state, was the reproduction of the metal. He had also tried the tests upon a decoction of onions (having learned that the deceased had eaten them for dinner on the day of her illness), and found that the precipitates were green and yellow, as stated by the preceding witnesses.

Mr. Luscombe, surgeon to the Exeter hospital, under whom the prisoner had studied, gave him an excellent character for skill and humanity. A number of other persons of respectability, resident in Falmouth, Foy, and other places in that neighbourhood, were also called for the same purpose. They all considered him a young man of great industry in his business, correctness in his morals, and gentleness in his disposition.

Mr. Justice Abbott then proceeded to sum up the whole case. After directing the attention of the Jury to the points for their consideration, he recapitulated the evidence on both sides. The charge alone occupied nearly three hours.

After consulting for about a quarter of an hour, the Jury returned a verdict of —*Not Guilty;* and it was received with evident marks of satisfaction by the greater part of the auditory.

The prisoner was a young man of a very mild and delicate appearance, and of a gentlemanly deportment: he was dressed in black, and during the whole trial, which occupied from eight in the morning until the same hour at night, conducted himself with becoming firmness. His imprisonment, since November last, had evidently affected his health.

---

The ensuing Course of Lectures at the Royal College of Surgeons, London, will commence on Monday the 5th of May, at four o'clock in the afternoon, and be continued on Mondays and Thursdays, at the same hour, until completed.—The Museum of the College will be open for inspection on Tuesday the 6th of May, and on Tuesdays and Fridays from such date until the last Friday in August. For particulars relating to admission, application is to be made at the College between the hours of twelve and three.

*Theatre of Anatomy, Hatton Garden.*—Mr. Taunton's Summer Course of Lectures on Anatomy, Physiology, Pathology, and Surgery, will commence on Saturday, May 24th, at eight o'clock in the evening, precisely, and be continued every Tuesday, Thursday, and Saturday, at the same hour.

Dr. Clutterbuck will begin his Summer Course of Lectures on the Theory and Practice of Physic, Materia Medica, and Chemistry, on Monday June the 2d, at ten o'clock in the morning.

In consideration of the personal benefits received from the professional talents of John Stevenson, Esquire, Great Russel Street, Bloomsbury Square, his Royal Highness the Duke of York has been pleased to appoint him his Surgeon-Oculist and Aurist.

Dr. Burrows has just published a new Book, containing "A Statement of Circumstances connected with the Apothecaries Act and its Administration."

Dr. A. P. Wilson Philip has in the press, which will speedily be published, "An Experimental Inquiry into the Laws of the Vital Functions, with some Observations on the Nature and Treatment of Internal Diseases." In part republished from the Philosophical Transactions, with the Report of the National Institute of France on the Experiments of M. Le Gallois, and Observations on that Report.

Deaths.—At Maidstone, Thomas Smith, M. D.—At Winborne, Dorsetshire, George Montague Sears, M. D.—Suddenly in consequence of Ossification of the Heart, Mr. Holman, Surgeon, of Crediton.—At Buckland, near Gosport, aged 105, Charles F. Gordon, Esq. late Surgeon of the Royal Hospital, Haslar.

